# Low-temperature synthesis and growth model of thin Mo_2_C crystals on indium

**DOI:** 10.1038/s41598-021-87660-7

**Published:** 2021-04-15

**Authors:** Omer Refet Caylan, Goknur Cambaz Buke

**Affiliations:** 1 Micro and Nanotechnology Graduate Program, Department of Materials Science and Nanotechnology Engineering, TOBB University of Economics and Technology, 06510 Ankara, Turkey; 2 National Nanotechnology Research Center, UNAM, Bilkent University, 06800 Ankara, Turkey

**Keywords:** Condensed-matter physics, Nanoscale materials

## Abstract

Chemical vapor deposition is a promising technique to produce Mo_2_C crystals with large area, controlled thickness, and reduced defect density. Typically, liquid Cu is used as a catalyst substrate; however, its high melting temperature (1085 °C) prompted research groups to search for alternatives. In this study, we report the synthesis of large-area thin Mo_2_C crystals at lower temperatures using liquid In, which is also advantageous with respect to the transfer process due to its facile etching. SEM, EDS, Raman spectroscopy, XPS, and XRD studies show that hexagonal Mo_2_C crystals, which are orthorhombic, grow along the [100] direction together with an amorphous carbon thin film on In. The growth mechanism is examined and discussed in detail, and a model is proposed. AFM studies agree well with the proposed model, showing that the vertical thickness of the Mo_2_C crystals decreases inversely with the thickness of In for a given reaction time.

## Introduction

With developments in the field of 2-dimensional materials and the demonstration of their novel properties^1–3^, the interest in transition metal carbides (TMCs) has increased again. These properties directly depend on the structure of the crystals, which can be controlled by processing. One of the most commonly used top-down methods to produce 2D TMCs is the selective etching of the “A” layer in the MAX phase^4^. In general, MXene flakes produced through this method have a very defective structure resulting from severe etching and functional groups originating from the wet chemistry^4^. Defective MXene flakes with functional groups may be very versatile for applications such as composites^5^ (as in graphene research); however, for the controlled growth of these crystals, alternative bottom-up approaches should be targeted.

Recently, with the focus on Mo_2_C, chemical vapor deposition (CVD) synthesis proved to be a very promising approach for achieving large area, controlled thickness, and reduced defect density^6^. In this method, a Cu foil on top of the Mo substrate (Mo-Cu stack) is heated. Above the melting temperature of Cu (1085 °C), it melts and covers the Mo substrate. When a Mo-Cu diffusion couple is formed at high temperature in an H_2_:CH_4_ environment, the steps that take place can be summarized as follows:

1. CH_4_ dissociates: CH_4_ → C + 2H_2_(g) (carbon flux)
2. Mo diffuses into Cu
3. Mo dissolves in Cu and forms an Mo–Cu alloy
4. Dissolved Mo atoms in Cu diffuse to the surface of Cu or the interface between Cu and Mo_2_C, where they meet carbon atoms,
5. Result: formation of 2Mo + C → Mo_2_C

To control the morphology of the crystals, several research groups successfully studied the effects of various processing parameters such as the thickness of Cu foil^7–9^, CH_4_:H_2_ ratio^6,8,10–13^, and duration^6,8,10–12,14^. Although temperature is a critical processing parameter in this process, its choice is very limited (above 1085 °C) because Cu must be in the liquid state to allow the diffusion of Mo atoms through Cu towards the surface.

To lower the CVD growth temperature of Mo_2_C crystals, alloys are used. For this purpose, Cahitoglu et al.^15^ employed a liquid bimetallic Sn–Cu alloy instead of Cu foil and showed that Mo_2_C could grow at temperatures as low as 880 °C with sizes in the range of μm in diameter and hundreds of nanometers in thickness. Sun et al.^16^ used Au, and recently, Young et al.^17^ suggested that Ag–Cu alloys could also be used for Mo_2_C synthesis at lower temperatures (1000 °C). Very recently, Young et al.^18^ studied the growth of thin Mo_2_C on In-Cu alloy and showed that increasing the In content decreases the alloy substrate melting temperature so that a lower temperature synthesis can be performed.

In the present work, only In was used to synthesize Mo_2_C at lower temperatures than Cu. In has a low carbon solubility, and its melting temperature is 150 °C. Another important advantage of In is its facile etching, which is particularly relevant during the transfer process.

## Results and discussion

### Synthesis and characterization of Mo_2_C crystals on In

**Figure 1:**
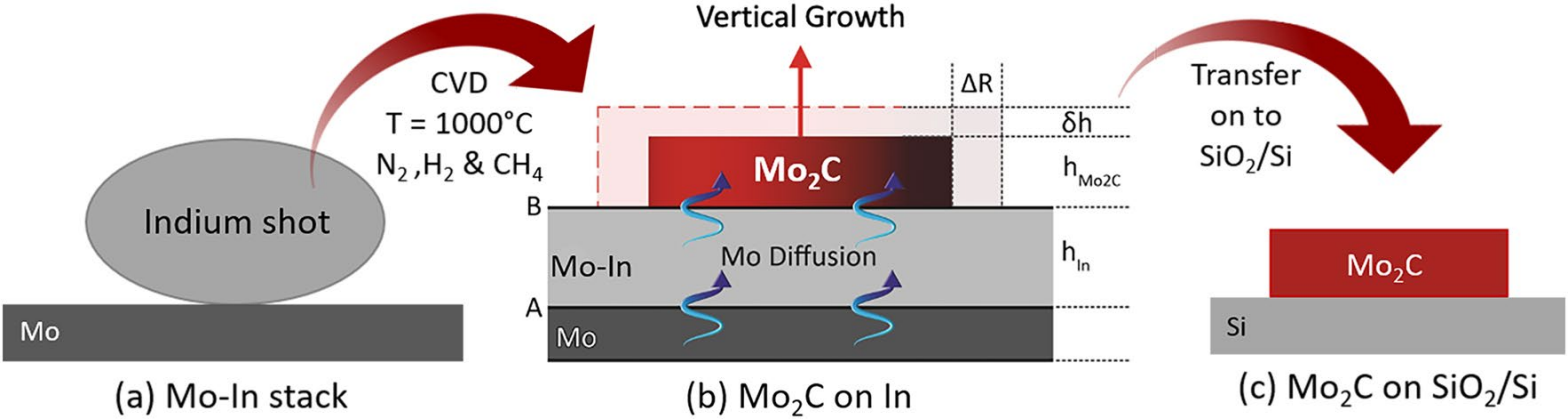
Illustration of the processing steps: (**a**) Mo-In stack, (**b**) Mo_2_C crystals growing at the In/Mo_2_C interface, and (**c**) Mo_2_C crystals transferred onto a SiO_2_/Si wafer.

An illustration of the processing steps is provided in Fig. 1. An In shot (120 mg) was placed on top of Mo foil (Mo–In stack in Fig. 1a) and heated up to 1000 °C under N_2_ and H_2_ flows. It was held at this temperature for 30 min in an N_2_:H_2_:CH_4_ environment for the growth of Mo_2_C crystals. Finally, the samples were cooled down to room temperature again under N_2_ and H_2_ flows.

In our study, because the hydrocarbon source attached to the system was CH_4_, the necessary high temperature for dissociation of methane lead to a lower limit for the synthesis temperature. We conducted the experiments at 850 °C, 900 °C and 1000 °C. However, at temperatures lower than 1000 °C, In did not fully wet the surface of the Mo foil; as a droplet it was not stable on Mo foil during the synthesis and the crystals formed at temperatures lower than 1000 °C were very small. This agrees well with the literature^15^. Young et al.^18^ also reported Mo_2_C flakes were small (< 1 μm) in the 800 °C synthesis despite a long duration (2 h). The reason is attributed to the increased viscosity of In-Cu alloy, which results in lower diffusion of Mo to the surface. Hence, in this study to be able to use the complementary characterization techniques on larger crystals, we did the systematical studies at 1000 °C to understand the mechanism.

**Figure 2:**
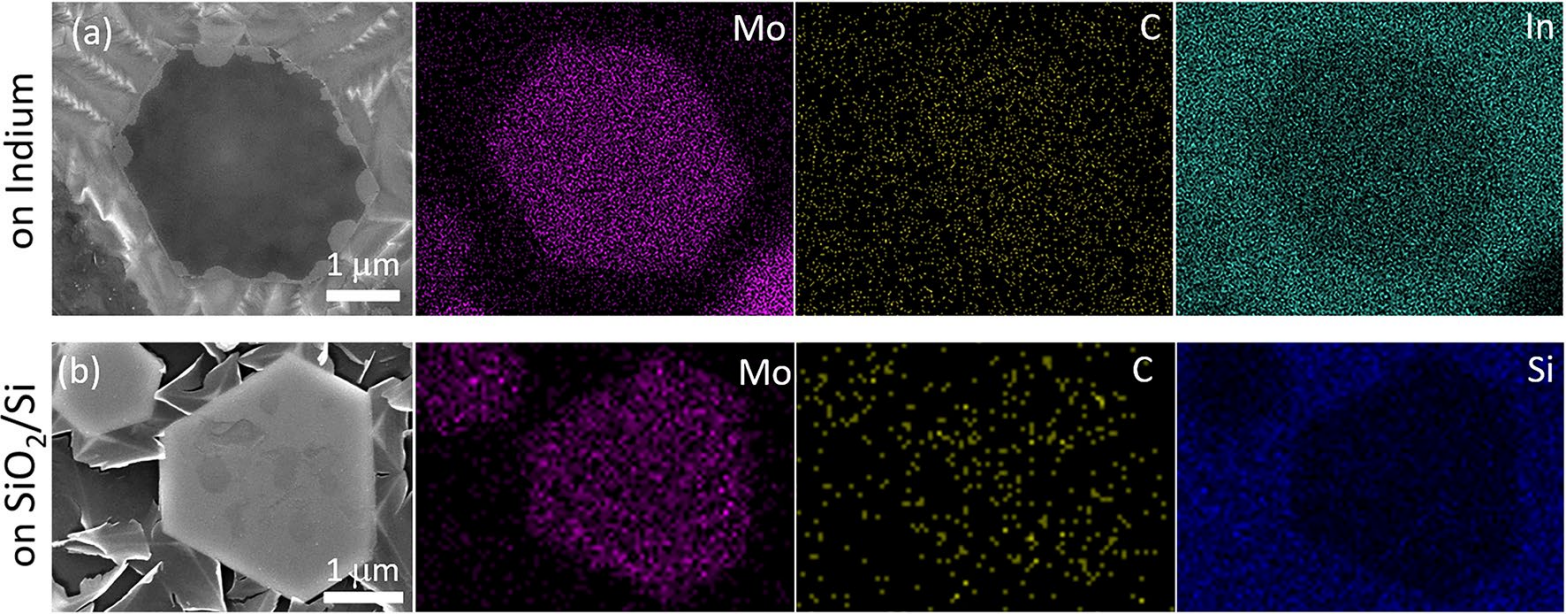
EDS maps from Mo_2_C (**a**) grown directly on In and (**b**) transferred to SiO_2_/Si wafer.

The composition and structure of the grown crystals were verified through complementary characterization techniques on as-grown crystals (on indium, Fig. 1b) and after transfer onto a SiO_2_/Si wafer (Fig. 1c). Figure 2 shows the EDS maps, which were taken from crystals present on In and SiO_2_/Si substrates (transferred). These results confirm that the crystals are composed of Mo and C.

**Figure 3:**
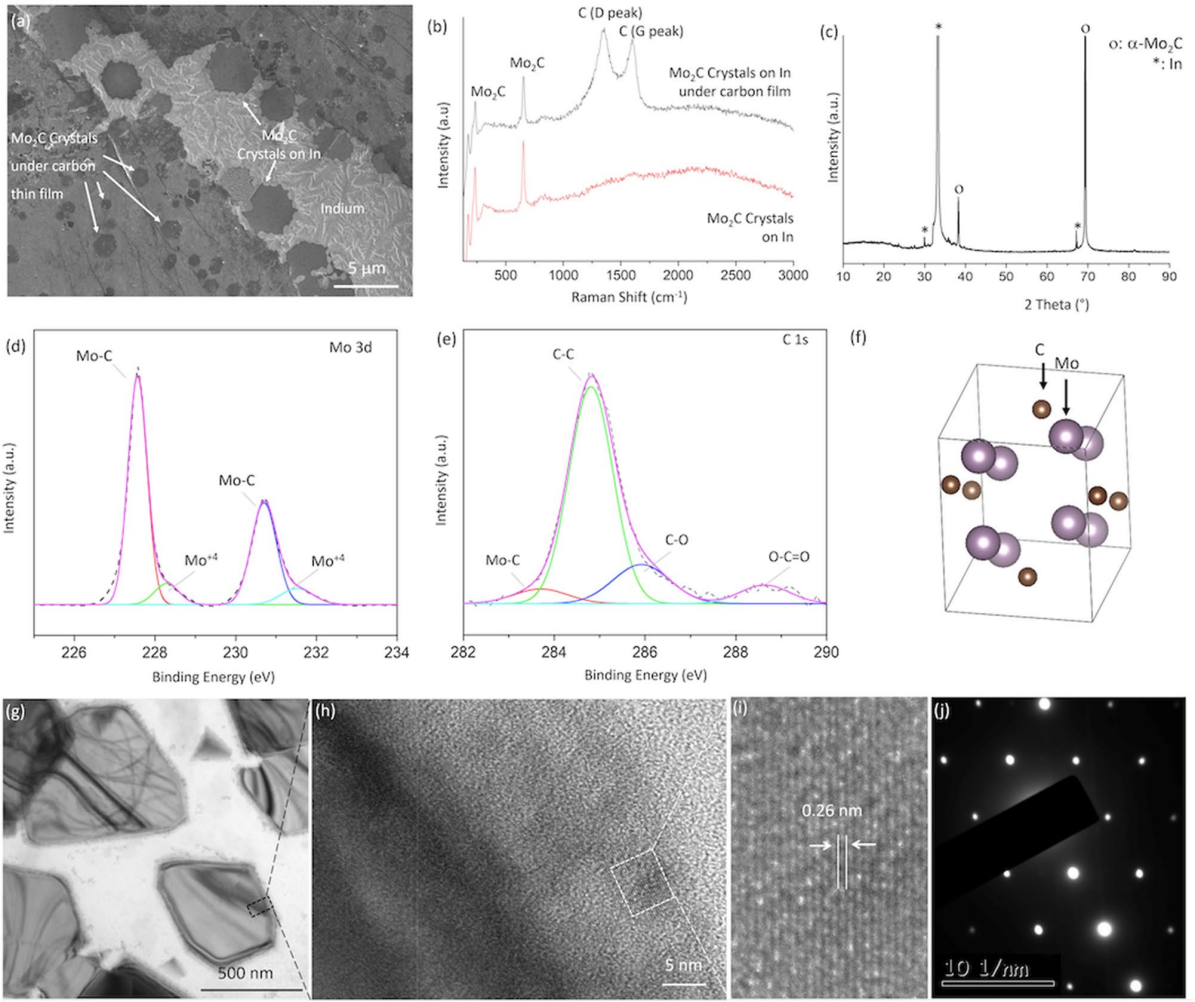
(**a**) SEM, (**b**) Raman spectroscopy, (**c**) XRD, (**d**,**e**) XPS, (**g**) Low resolution and (**h**,**i**) high resolution TEM images, (**j**) SAED studies performed on the Mo_2_C crystals synthesized on 120 mg In at 1000 °C; t = 30 min under N_2_:H_2_:CH_4_ (100:50:5) flow; (**f**) the unit cell of orthorhombic Mo_2_C crystal drawn using VESTA.

SEM and Raman spectroscopy studies (Fig. 3) performed on Mo_2_C crystals as-grown on In show that during the crystal growth, a carbon thin film is also formed on the liquid In surface. Hence, some of the Mo_2_C crystals became located under the carbon thin film and some on bare In (Fig. 3a). XPS results corroborated the formation of Mo_2_C crystals and the carbon thin film (Fig. 3c). Raman spectroscopy results (Fig. 3b), which were obtained from the crystals in both regions (under carbon thin film and on bare In surface), confirmed that the grown crystals are Mo_2_C and the thin film is mostly amorphous carbon. The crystal structure of the Mo_2_C crystals is investigated and through Raman Spectroscopy analysis^9^ (Raman peaks at ∼ 231, and 656 cm^−1^), XRD (2 theta at 38° and 69°) and TEM (Fig. 3g) studies it is found to be orthorhombic which also agrees well with literature^6,8–10,12,13,15,18–23^. Figure 3h,i exhibit the high-resolution transmission electron microscopy (HRTEM) image taken from the edge of a crystal (Fig. 3g). The average spacing of lattice fringes is 0.26 nm which is consistent with (200) interplanar spacing of α-Mo_2_C that can be seen at 38° in XRD pattern (Fig. 3c). The SAED pattern given in Fig. 3j confirms that thin crystals are highly crystalline.

The SEM micrograph in Fig. 3a shows that the Mo_2_C crystals, with or without carbon thin film, are mostly hexagonal in shape. However, the crystals under the carbon thin film are smaller than those of on bare In. This difference can be explained by the different diffusion mechanisms of Mo atoms on the In surface. In the presence of a carbon thin film, the diffusion of the Mo atoms takes place through the interface between the In surface and the amorphous carbon blanket by the relaxed vacancy exchange mechanism, which is much slower than the diffusion of Mo adatoms on the bare In surface by hopping motions^24^.

### Growth mechanism of Mo_2_C crystals through CVD

When a Mo-In stack is heated and held at 1000 °C in an N_2_:H_2_:CH_4_ environment, first In melts and covers the entire Mo substrate; then, Mo dissolves in In and forms an Mo-In alloy. At steady-state regime, the dissolved Mo atoms are subjected to a chemical driving force due to the concentration difference associated with the solubilities at the adjacent phase boundaries, namely Mo/MoIn (shown with A in Fig. 1b) and MoIn/Mo_2_C (shown with B in Fig. 1b). This driving force is inversely proportional to the thickness of the In layer and linearly proportional to the solubility differences. Under this driving force, the dissolved Mo atoms form a steady flux at the interface between the Mo–In alloy (A) and Mo_2_C layer (B). Mo atoms travel to the Mo_2_C/MoIn interface through MoIn by vacancy mechanism and meet the incoming flux of carbon atoms to form Mo_2_C.

**Figure 4:**
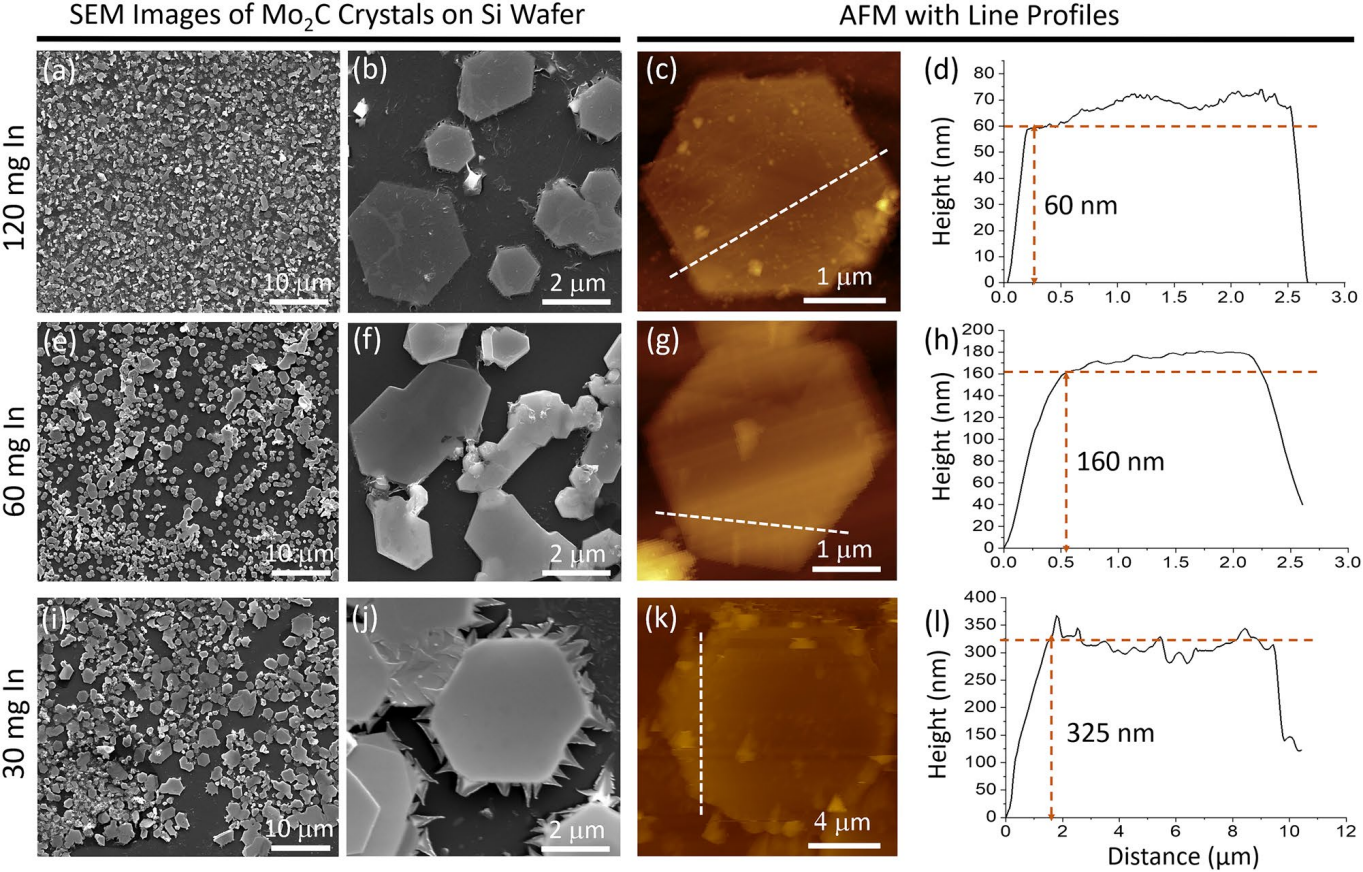
SEM, AFM images and corresponding height profiles of Mo_2_C crystals grown on (**a**–**d**) 120-mg, (**e**–**h**) 60-mg, and (**i**–**l**) 30-mg In.

Carbon atoms come first from the dissociation of CH_4_ at the Mo_2_C crystal surface, exposed to the environment. Then they penetrate and diffuse through the Mo_2_C crystal and reach to the reaction interface between Mo_2_C and MoIn (shown with B in Fig. 1b) where chemical combination takes place between carbon and Mo atoms incoming from MoIn solution to form a fresh new Mo_2_C layer. Carbon probably diffuses by an interstitial mechanism in Mo_2_C^25^, and the activation energy for diffusion involves only the thermally activated atomic hopping motion (motional enthalpy). Moreover, it can be seen from the unit cell (Fig. 4b) that half of the octahedral sites are occupied by the carbon atoms, and the remaining sites are empty. This also makes carbon diffusion easy. On the other hand, Mo diffuses in Mo_2_C by vacancy diffusion mechanism, hence the activation energy for the diffusion is composed of two parts: related to vacancy formation and atomic jump motion. As a result, the diffusion flow of carbon atoms through the Mo_2_C layer, via hopping motion along the interstitial sites, may be extremely fast compared to the opposite diffusion of Mo atoms in the same structure through the thermally generated Mo vacancies, which otherwise may be combined with carbon at the surface of Mo_2_C crystals to form fresh new layers^26^.

### Growth model for the Mo_2_C crystals through CVD

To model the growth of the crystal at steady-state regime, volume conservation can be used. That is, the total volume of Mo atoms attaching to the Mo_2_C crystal can give the total volume expansion of the crystal (because carbon atoms, which occupy half of the octahedral sides, are too small, and their effect on the volume expansion is negligible). The total volume of Mo atoms attaching to the Mo_2_C crystal can be found from the Mo flux at the In–Mo_2_C interface.

In this system, after a short transient regime, the system reaches a steady state in which the diffusion flux is not a function of time. The driving force for Mo diffusion from A to B is the concentration gradient between points A and B, so the flux between A and B can be written as follows:

1 $${{\rm J}} = {{\rm D}} \times \frac{{\left( {{{\rm C}}_{{\rm{A}}}^{Mo} - {{\rm C}}_{{\rm{B}}}^{Mo}} \right)}}{{{{\rm h}}_{{{\rm{In}}}} }}$$

where D is the diffusivity of Mo in In, and C_A_ and C_B_ are the concentrations of Mo (i.e., equilibrium solubilities of Mo with respect to adjacent phases) at points A and B, respectively. From the definition of flux, the number of Mo atoms coming from the Mo-In alloy to the In- Mo_2_C interface for a given time interval $\Delta {{\rm t}}$ can be found from the following expression:

2 $$\# \;{{\rm of}}\;{{\rm Mo}}\;{{\rm atoms}}\;{{\rm entering}}\;{{\rm crystal}} = {{\rm J}} \times {{\rm A }} \times \Delta {{\rm t}}$$

where A is the area and Δt is the duration. In this model, the Mo_2_C crystal is modeled as a cylinder, that is, A = π · R^2^. Therefore, the total volume change can be written as follows:

3 $$\Delta {{\rm V}}_{{\rm Total}} = {{(\# }}\;{{\rm of}}\;{{\rm Mo}}\;{{\rm atoms}}\;{{\rm entering}}\;{{\rm crystal)}} \times {{\rm (V}}_{{{{\rm Mo}}}} {)}$$

here V_Mo_ is the specific volume of one Mo atom. With the addition of Mo atoms, the cylinder grows in both the lateral and vertical directions. By substituting Eq. (2) into Eq. (3), we obtain:

4 $$\Delta {{\rm V}}_{{\rm Lateral}} + \Delta {{\rm V}}_{{\rm Vertical}} = {{\rm (J}} \times {{\rm A}} \times \Delta {{\rm t )}} \times {{\rm (V}}_{{\rm Mo}})$$

5 $$\Delta {{\rm V}}_{{\rm Lateral}} = {(2}\uppi {{\rm R}} \times \Delta {{\rm R}} \times h_{Mo_{2}C} {)}$$

6 $$\Delta {{\rm V}}_{{\rm Vertical}} = {(}\uppi {{\rm R^{2}}} \times\updelta h_{Mo_{2}C}{)}{{\rm}}$$

Substituting Eqs. (1), (5), and (6) into Eq. (4), we obtain

7 $${(2}\uppi {{\rm R}} \times \Delta {{\rm R}} \times h_{Mo_{2}C} {)} + {(}\uppi {{\rm R^{2}}} \times\updelta h_{Mo_{2}C} {)} = {{\rm (J}} \times\uppi {{\rm R^{2}}} \times \Delta {{\rm t)}} \times {{\rm (V}}_{Mo})$$

Dividing Eq. (7) by (π × R^2^) gives:

8 $$\left( {\frac{{2 \times \Delta R \times h_{Mo_{2}C}}}{R}} \right){ + (}\updelta \;{{\rm h)}} = {{\rm (J}} \times \Delta {{\rm t)}} \times {{\rm (V}}_{{\rm Mo}})$$

To investigate the vertical growth, one can assume that there is no lateral growth (ΔR = 0); then Eq. (8) gives:

9 $${ }\mathop \smallint \limits_{{h_{0} }}^{h} dh = \int\limits_{0}^{t} {{{\rm J}} \times {{\rm (V}}_{{\rm Mo}}) \times {{\rm dt}}} \to {{\rm h}} = {{\rm J}} \times {{\rm (V}}_{{\rm Mo}}) \times {{\rm t}} + h_{o}$$

By substituting Eq. (1) into Eq. (9) for the vertical growth, we obtain:

10 $$h_{Mo_{2}C} = {{\rm D}} \times \frac{{\left( {{{\rm C}}_{{{\rm A}}}^{Mo} - {{\rm C}}_{{{\rm B}}}^{Mo} } \right)}}{{{{\rm h}}_{{{{\rm In}}}} }} \times {{\rm (V}}_{{\rm Mo}}) \times {{\rm t + }}h_{o}$$

To test this model, Mo_2_C crystals grown on various amounts of In (120 mg, 100 mg, 60 mg, and 30 mg) were compared. Because In wets the Mo surface at this temperature, the In amount can be directly related to the thickness of the In layer. Note from Fig. 4 that as the amount of In (layer thickness) increases, the thickness of the Mo_2_C crystals decreases. This is also in agreement with previous studies performed with Cu^7–9^.

**Figure 5:**
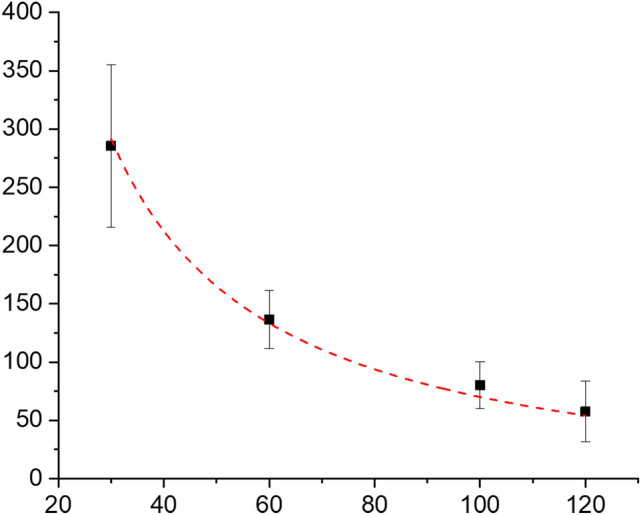
Mo_2_C crystal thickness dependence on the In amount (the red line represents the model and the data points represent the AFM measurements).

To compare the proposed model with experimental results, AFM measurements were performed on several crystals for various In amounts. Figure 5 shows the results of the experimental measurements together with those from the model (Eq. 10). Note that the vertical growth of the crystals clearly decreases inversely with the thickness of In for a given reaction time t.

## Conclusions

In this study, we showed that In may be an ideal substrate for growing Mo_2_C crystals via CVD because it enables the formation of high quality, large-area, thin Mo_2_C crystals at lower temperatures (1000 °C) than copper (1085 °C); and its facile etching makes the transfer process easy. Complementary characterization studies showed that hexagonal-shaped, large-area, thin Mo_2_C crystals, which are orthorhombic, grow along the [200] direction with an amorphous carbon thin film. The Mo_2_C crystals, grown under the carbon thin film, are found to be smaller and thinner than those formed on bare In surface as expected. The growth mechanism of Mo_2_C crystals through CVD was examined in detail, and a model was proposed showing that the vertical growth of the Mo_2_C crystals decreases inversely with the thickness of the In. This model was verified by AFM studies.

With further optimization, the synthesis temperature may be lowered; however, our studies show that, in this type of process, it is not only the catalyst (In) melting point that is critical. While choosing the catalyst material and determining the temperature, one needs to consider the wetting of the catalyst and the diffusivity of Mo in catalyst (viscosity of the catalyst) together with the decomposition of the hydrocarbon gas.

## Materials and methods

### Mo_2_C synthesis

Molybdenum foils (Nanografi, NG06BPM0190P1, 0.1 mm thick, 99.95% purity, 10 mm diameter) were sonicated in 1 M 50 mL hydrochloric acid solution (36.5–38%, Sigma Alrich, 07102), then vigorously stirred in deionized water and ethanol for 10 min each, and finally dried with N_2_ gas. Indium shots (Alfa Aesar, 11026, 99.9% purity) were cut according to a prescribed weight and placed on top of Mo foils. Samples were then placed on top of a quartz crucible and then in a mass-flow-controlled (Beijing Sevenstar Electronics Co., Ltd) atmospheric-pressure CVD furnace (Protherm, STF13/50/300) with a 110 cm-long, 50 mm-diameter quartz tube. This tube was purged with N_2_ gas for at least 10 min before heating. The substrates were heated to 1000 °C for 30 min under 100 sccm N_2_ and 50 sccm H_2_ flows. Once the growth temperature (1000 °C) was reached, a 5 sccm CH_4_ flow was introduced for 30 min. After the growth was finished, the samples were pushed out of the hot zone at 950 °C while maintaining N_2_ and H_2_ flows at the same level until the sample temperatures reached a temperature below 50 °C; this took approximately 1 h.

### Transfer of Mo_2_C crystals

The transfer of these crystals was achieved by applying approximately 1 mL of cellulose nitrate solution on the sample surface. Then, the sample was left to dry off for 20 min to form a film, which was then placed in a 1 M (NH_4_)_2_S_2_O_8_ solution at RT to etch away the In. The etching process takes from 30 min to 2 h depending on the amount of In used in the experiment. After etching the In layer, the film with crystals started swimming in the solution (depending on the applied solution amount) and then rinsed with DI water for 10 min to wash away the etchant. The film was transferred onto target substrates by the fishing method, and after drying overnight, it was submerged in acetone. The simplicity of this method is that the film obtained after drying the cellulose nitrate solution is not as delicate as PMMA coatings, and it is easily removable by acetone. There are several reports that mention the residues PMMA leaves^27–29^. Such residues were not observed with this method.

### Characterization

Optical images were obtained using a Nikon Eclipse LV150N microscope. The morphology of the Mo_2_C crystals synthesized on In was identified via SEM equipped with electron dispersive spectroscopy (EDS) (FEI Quanta 200 FEG). The thickness measurements of the crystals were measured using AFM in tapping mode (Park XE-100 AFM, Park Systems). The carbon structures and Mo_2_C crystals were studied and identified using Raman spectroscopy (Witec Alpha300S with an excitation wavelength of 532 nm) and X-ray photoelectron spectroscopy (XPS) (K-Alpha Model XPS spectrometer, Thermo Fisher Scientific, UK; Al Kα radiation, 1486.6 eV was employed as the X-ray source). X-ray diffraction (XRD) was used to analyze the phase of the Mo_2_C crystals (D8 Advance Bruker with CuKα radiation). Crystal structure drawings were produced by VESTA^30^.
